# Supplementary material for: Distinct neural representations during a brain–machine interface and manual reaching task in motor cortex, prefrontal cortex, and striatum
Source: Sci Rep. 2023 Oct 19;13:17810. doi: 10.1038/s41598-023-44405-y (PMC10587077; doi:10.1038/s41598-023-44405-y)
Supplement: Supplementary file 1 — Supplementary Information. [file 41598_2023_44405_MOESM1_ESM.pdf]

Supplementary Information

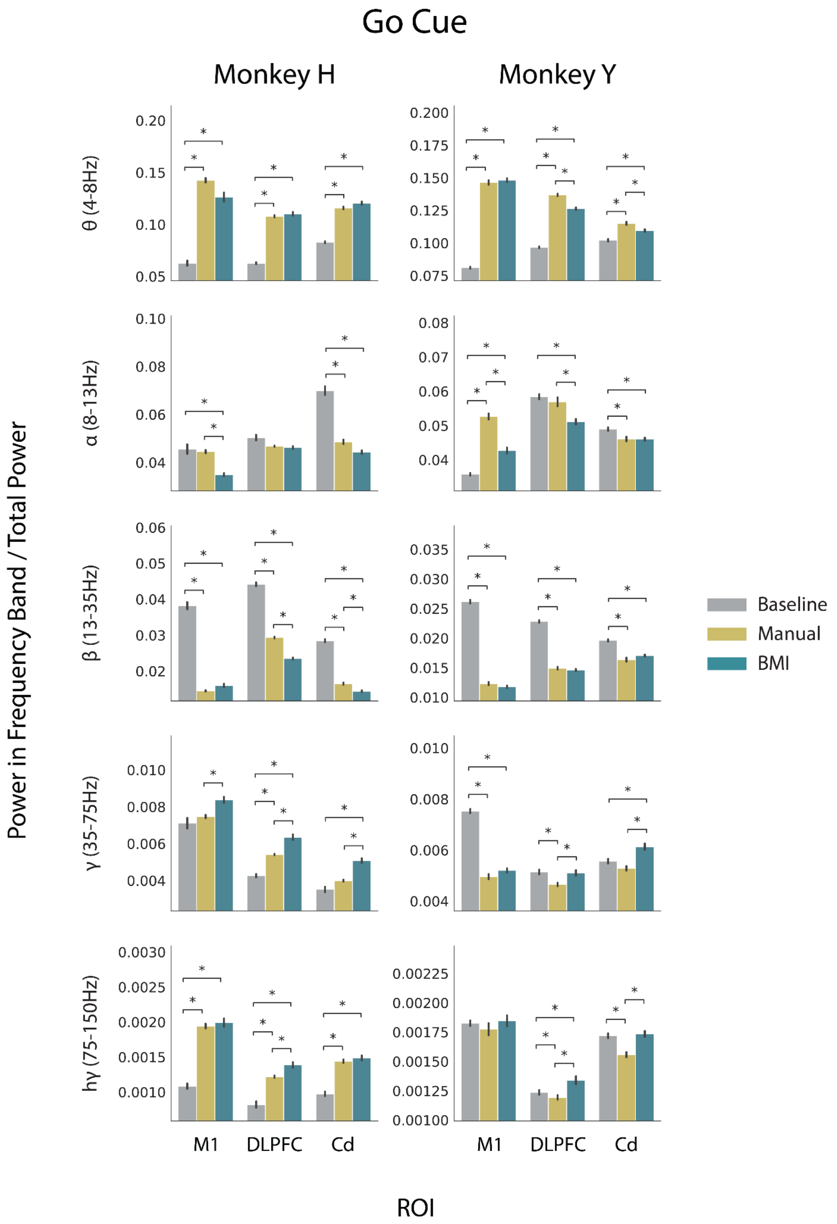

Supplementary Figure 1. Spectral power in M1, DLPFC, and Cd during baseline, manual control, and BMI control at the go cue. LFP was decomposed into 5 distinct frequency bands. The average power normalized to total power in each of these bands in each ROI was compared during baseline (gray), manual (yellow), and BMI (teal) at the go cue for Monkey H (left) and Monkey Y (right). Error bars represent standard error mean across days. Normalized spectral power estimates significantly differing between task types after Bonferroni correction for multiple comparisons are indicated with an asterisk (Supplementary Table 1, Bonferroni corrected p-value < 0.05).

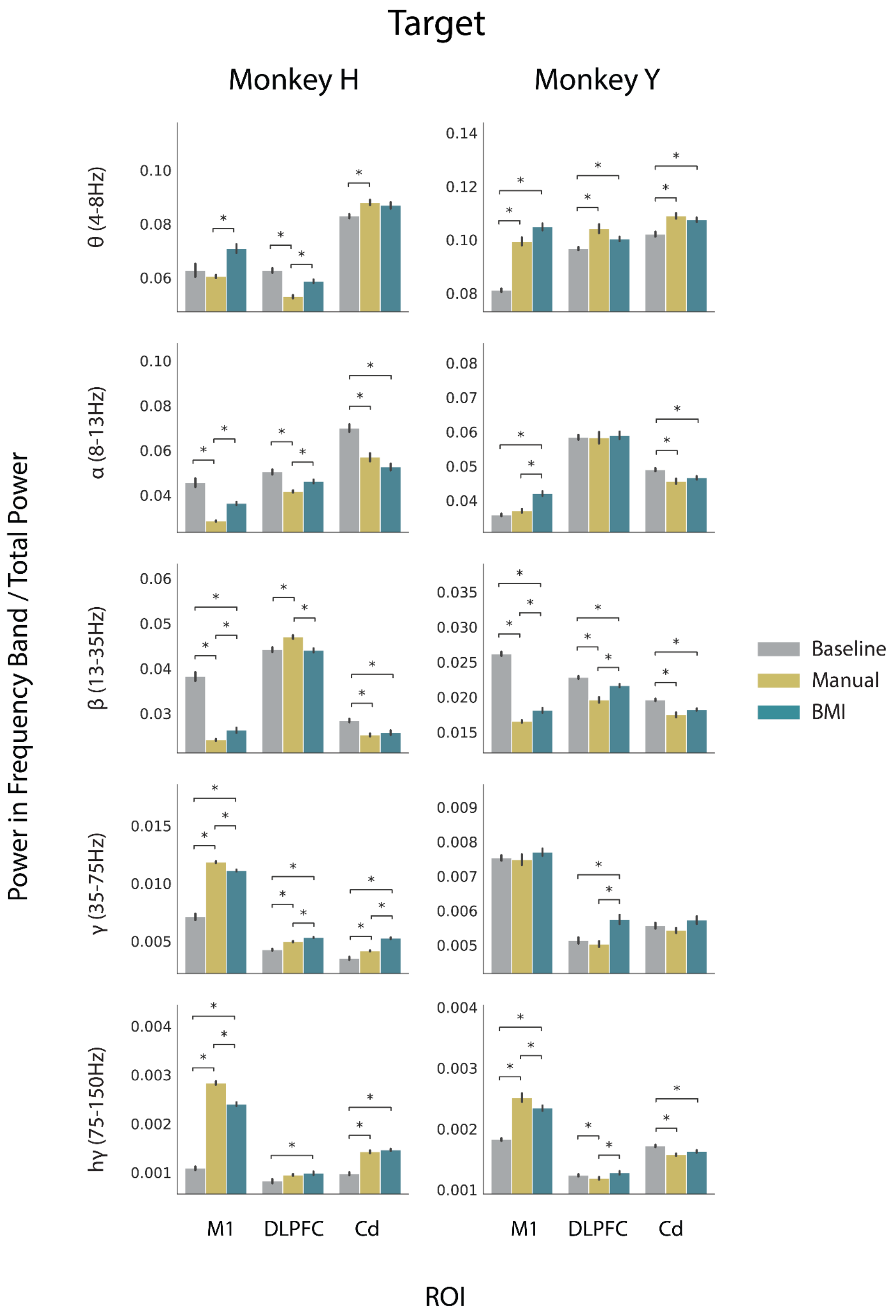

Supplementary Figure 2. Spectral power in M1, DLPFC, and Cd during baseline, manual control, and BMI control at target acquisition. LFP was decomposed into 5 distinct frequency bands. The average power normalized to total power in each of these bands in each ROI was compared during baseline (gray), manual (yellow), and BMI (teal) at target acquisition Monkey H (left) and Monkey Y (right). Error bars represent standard error mean across days. Normalized spectral power estimates significantly differing between task types after Bonferroni correction for multiple comparisons are indicated with an asterisk (Supplementary Table 2, Bonferroni corrected p-value < 0.05).

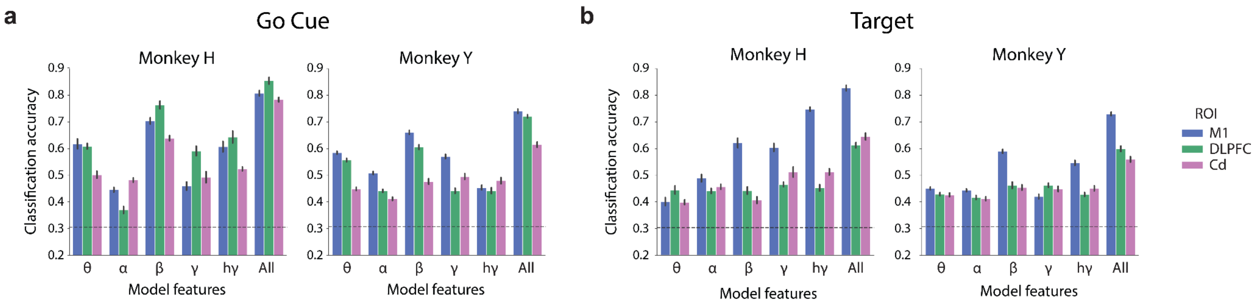

Supplementary Figure 3. Task-type classification accuracy is above chance for all individual frequency bands and highest for the combination of all frequency bands in all ROIs. (a) Mean 10-fold cross-validated classification accuracy across days for a *3-class* task-type QDA classifier trained to distinguish between BMI control, manual control, and baseline using individual frequency bands and all combined bands at the go cue for Monkey H (left) and Monkey Y (right). Chance accuracy shown as a dashed line. Error bars represent standard error of the mean across days. (b) Same as (a) but for target acquisition, rather than go cue.

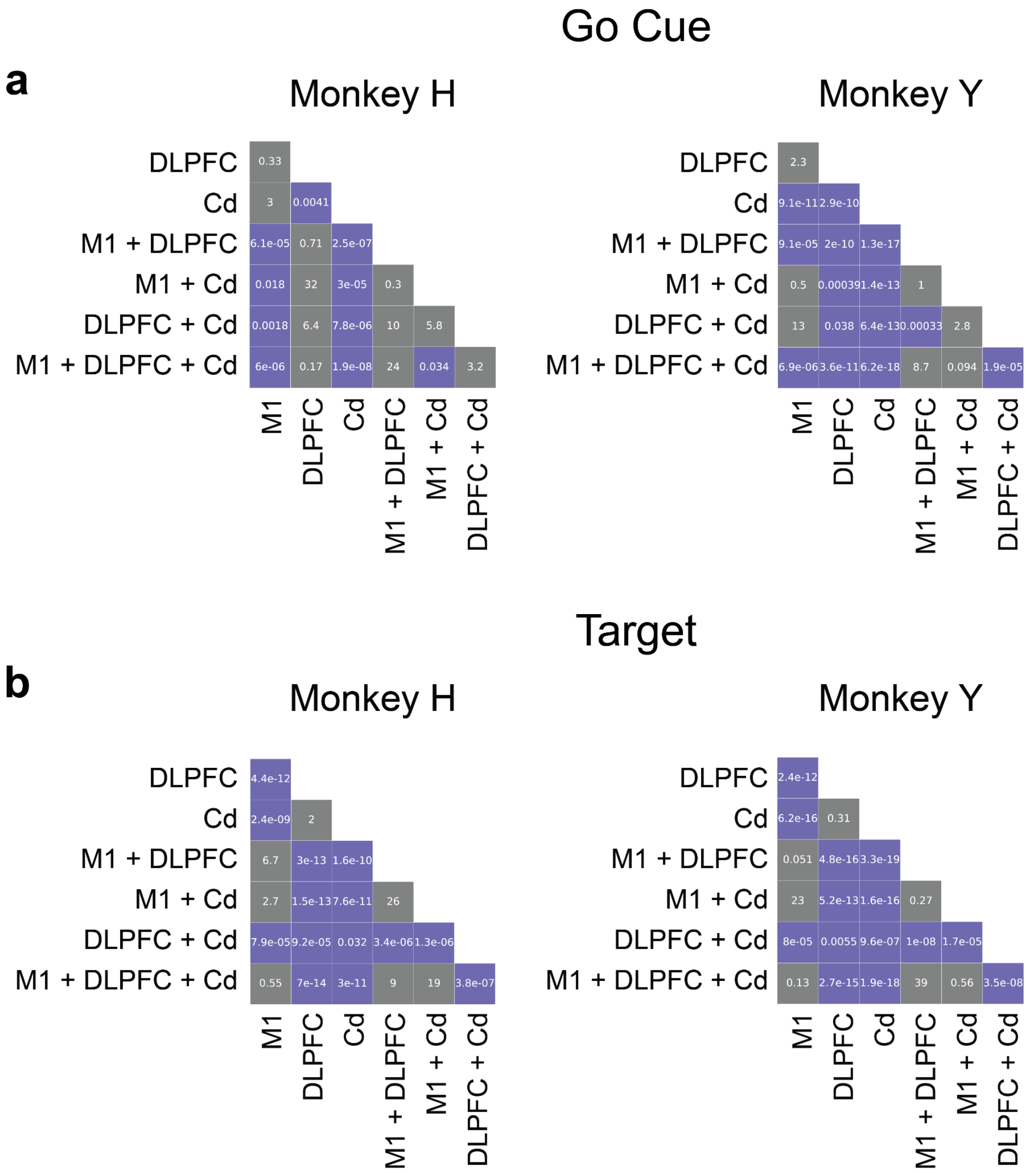

Supplementary Figure 4. Statistical significance values for pairwise comparisons of all *3-class* task-type QDAmodels. (a) Bonferroni corrected p-values for all unpaired t-tests comparing cross-validated classification accuracy obtained from QDA classifiers with input from different combinations of ROIs at the go cue for Monkey H (left) and Monkey Y (right). Purple squares indicate a Bonferroni corrected p-value < 0.05. Gray squares indicate a Bonferroni corrected p-value > 0.05. (b) Same as (a) but for features obtained at target acquisition, rather than at go cue.

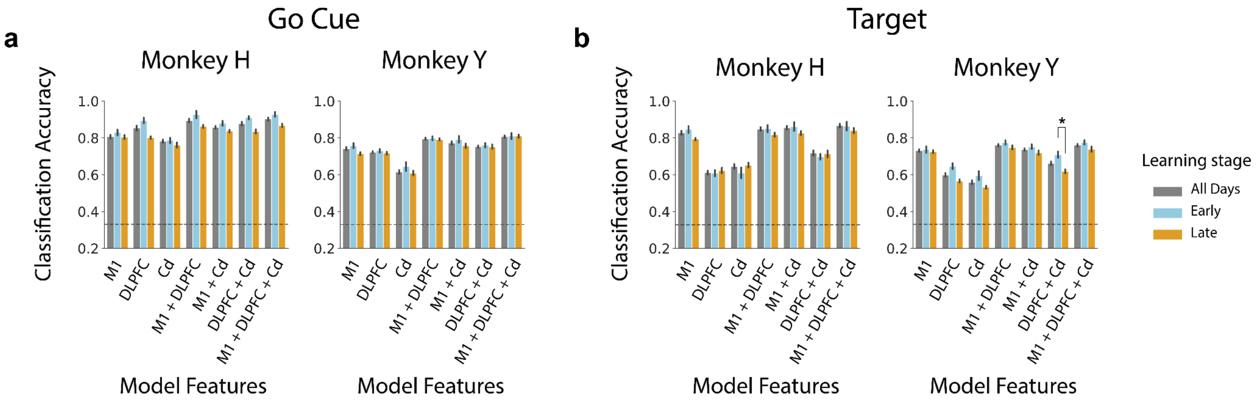

Supplementary Figure 5. Classification accuracy using the *3-class* task-type QDA model is similar across BMI learning stages. (a) Mean 10-fold cross-validated classification accuracy across all days (gray), early learning days (blue), and late learning days (orange) for a *3-class* task-type QDA classifier trained to distinguish between BMI control, manual control, and baseline using all frequency bands from individual ROIs or combinations of ROIs at the go cue for Monkey H (left) and Monkey Y (right). Chance accuracy shown as a dashed line. Error bars represent standard error mean across days. (b) Same as (a) but for target acquisition, rather than go cue.

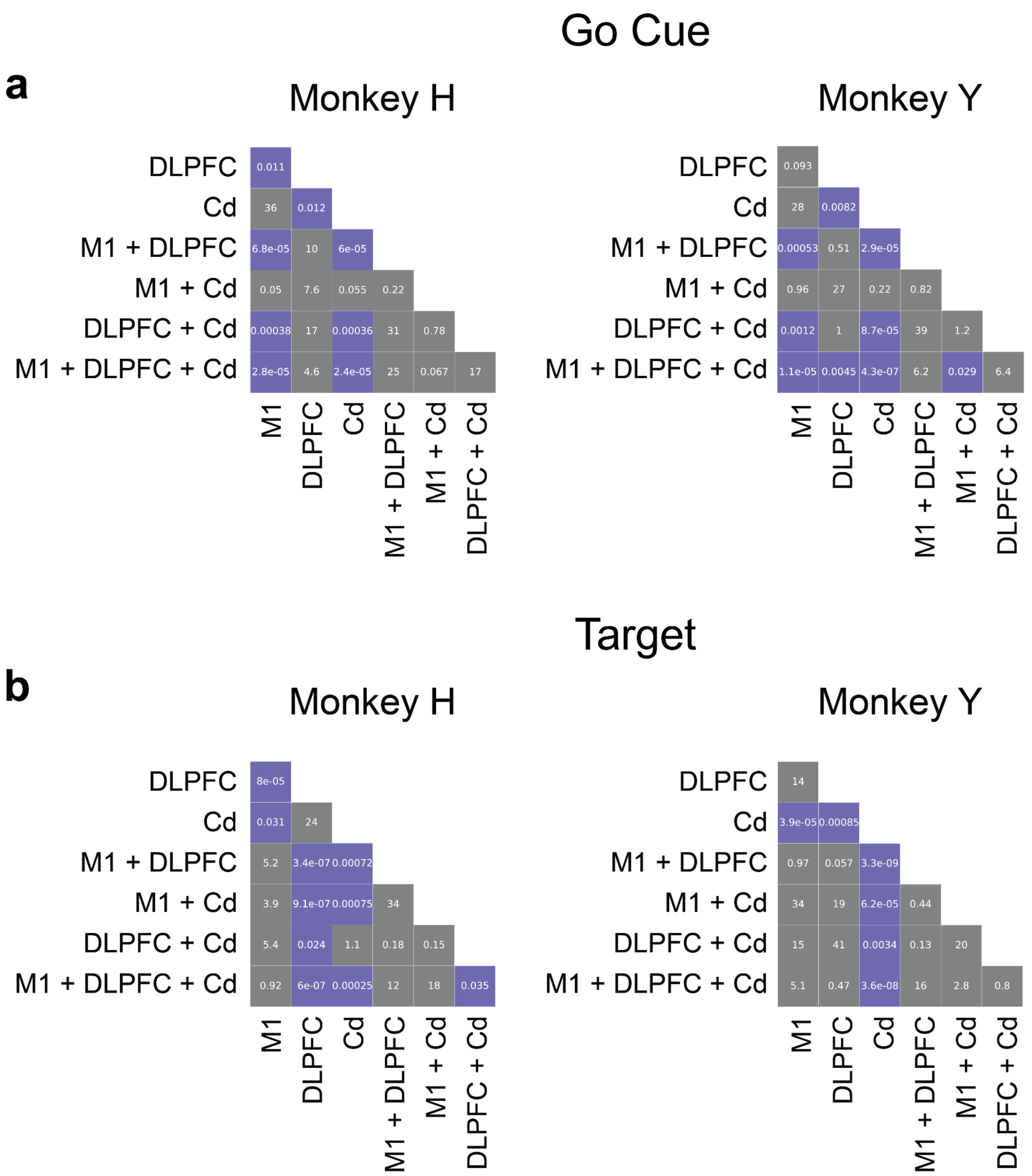

Supplementary Figure 6. Statistical significance values for pairwise comparisons of all *2-class* task-type QDA models. (a) Bonferroni corrected p-values for all unpaired t-tests comparing cross-validated classification accuracy obtained from QDA classifiers with input from different combinations of ROIs at the go cue for Monkey H (left) and Monkey Y (right). Purple squares indicate a Bonferroni corrected p-value < 0.05. Gray squares indicate a Bonferroni corrected p-value > 0.05. (b) Same as (a) but for features obtained at target acquisition, rather than at go cue.

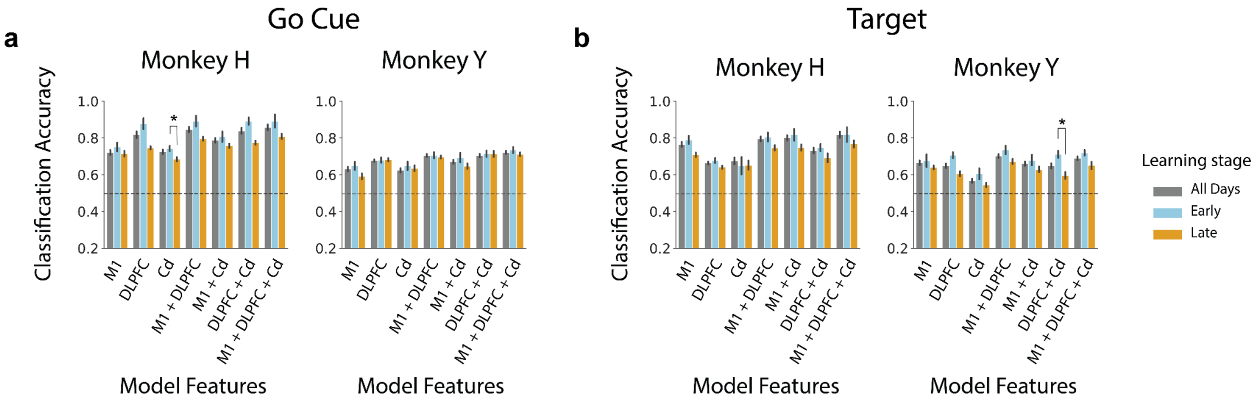

Supplementary Figure 7. Classification accuracy using the *2-class* task-type QDA model is similar across BMI learning stages. (a) *Mean 10-fold cross-validated classification accuracy across all days (gray), early learning days (blue), and late learning days (orange) for a 2-class task-type QDA classifier trained to distinguish between BMI and manual control using all frequency bands from individual ROIs or combinations of ROIs at the go cue for Monkey H (left) and Monkey Y (right). Chance accuracy shown as a dashed line. Error bars represent standard error mean across days. (b) Same as (a) but for target acquisition, rather than go cue.*

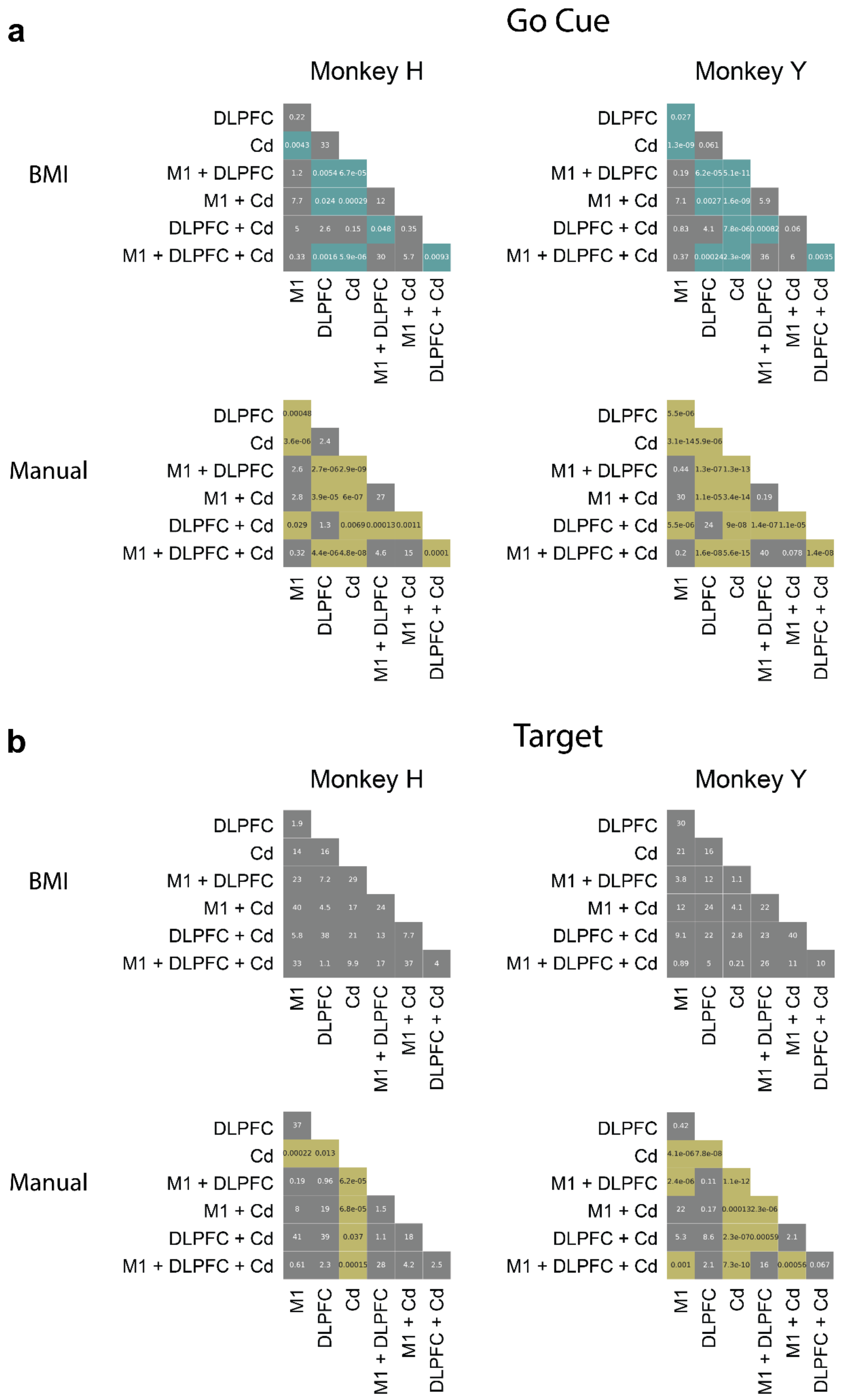

Supplementary Figure 8. Statistical significance values for pairwise comparisons of all *8-class* target-direction LDA models. (a) Bonferroni corrected p-values for all unpaired t-tests comparing cross-validated classification accuracy obtained from LDA classifiers with input from different combinations of ROIs at the go cue for Monkey H (left) and Monkey Y (right), under BMI control (top, teal) and under manual control (bottom, gold). Teal or gold squares indicate a Bonferroni corrected p-value < 0.05. Gray squares indicate a Bonferroni corrected p-value > 0.05. (b) Same as (a) but for features obtained at target acquisition, rather than at go cue.

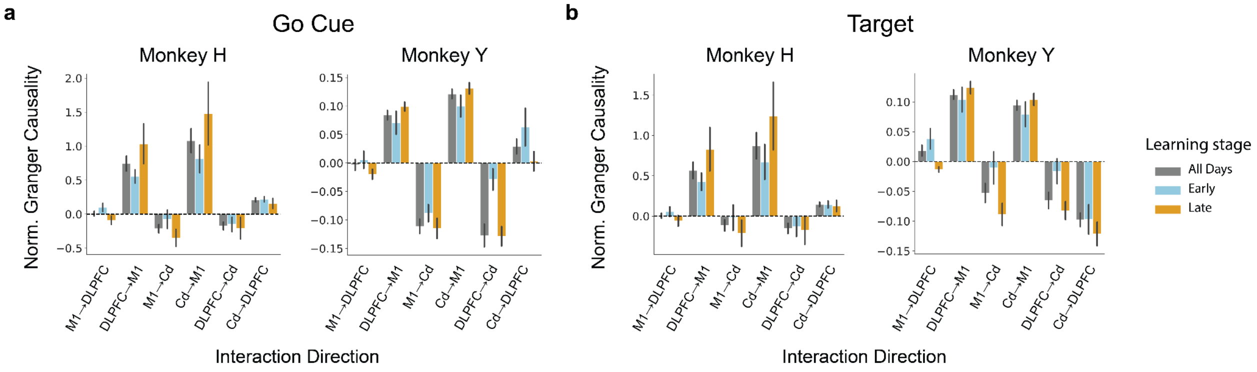

Supplementary Figure 9. Normalized granger causality is similar across BMI learning stages (a) Normalized Granger causality estimates at the go cue during BMI control across all days (gray), early BMI learning (blue), or late BMI learning (orange). Error bars represent standard error mean across days. (b) Same as (a) but for target acquisition, rather than go cue.

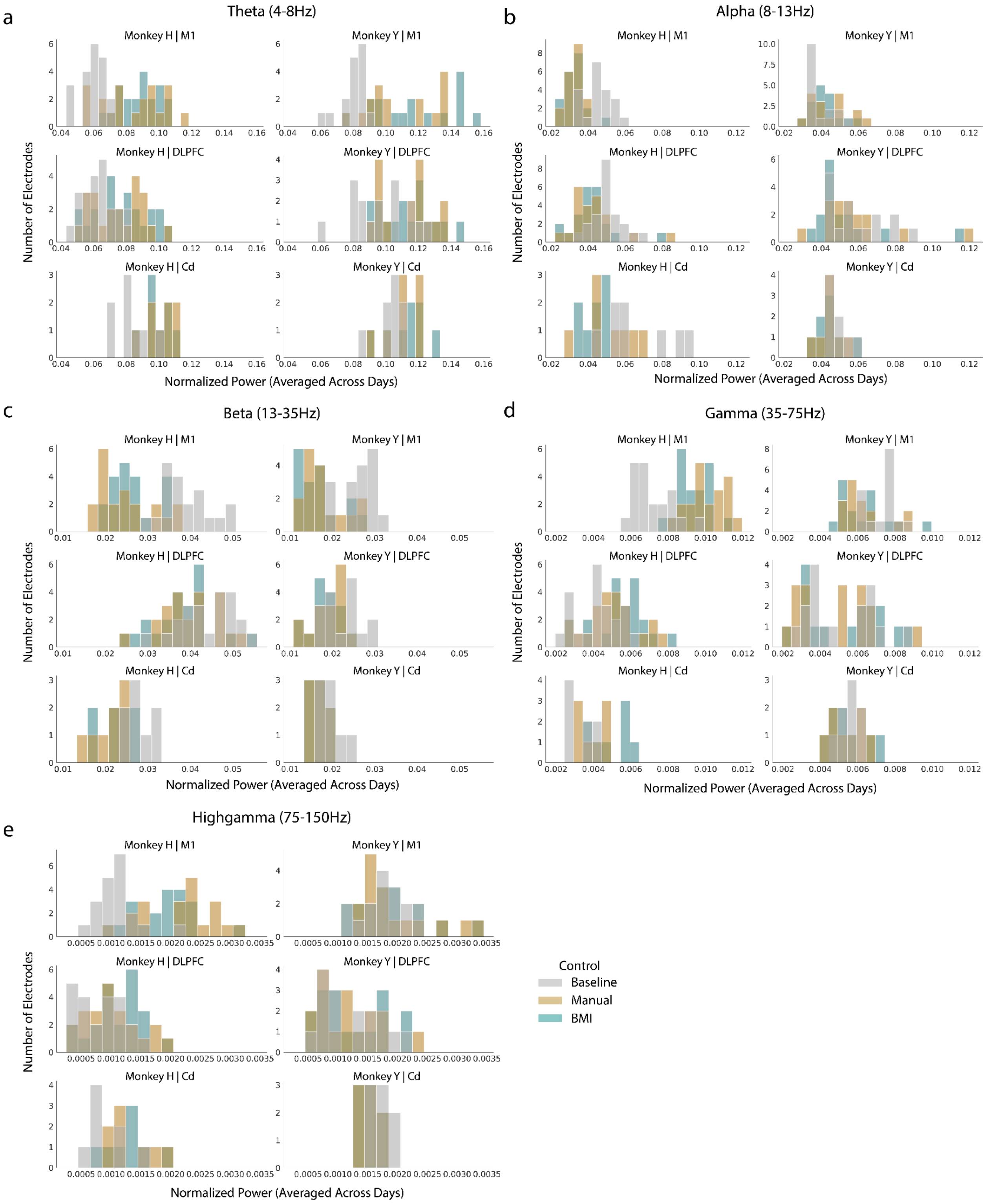

Supplementary Figure 10. Distributions of average normalized power for each electrode in M1 (top), DLPFC (middle), and Cd (bottom) for (a) theta, (b) alpha, (c) beta, (d) gamma, and (e) highgamma for Monkey H (left) and Monkey Y (right) during baseline (gray), manual (yellow), and BMI (teal).

Go Cue: Comparing Average Power Between Task Types

			M1		DLPFC		Cd	
**Subject**	**Frequency Band**	**Comparison**	**t-statistic**	**Bonferroni corrected p**	**t-statistic**	**Bonferroni corrected p**	**t-statistic**	**Bonferroni corrected p**
Monkey H	Theta (4-8Hz)	Baseline vs Manual	**-24.496**	**5.816e-10***	**-31.148**	**3.393e-11***	**-29.790**	**5.758e-11***
		Baseline vs BMI	**-12.170**	**1.860e-06***	**-23.000**	**1.221e-09***	**-20.825**	**3.913e-09***
		Manual vs BMI	3.816	0.110532	-1.461	7.633671	-3.380	0.246192
	Alpha (8-13Hz)	Baseline vs Manual	0.507	27.953291	3.006	0.492121	**11.515**	**3.441e-06***
		Baseline vs BMI	**5.242**	**9.317e-03***	3.798	0.114247	**13.665**	**5.055e-07***
		Manual vs BMI	**11.801**	**2.622e-06***	0.835	18.898263	3.964	0.084514
	Beta (13-35Hz)	Baseline vs Manual	**24.787**	**5.059e-10***	**23.012**	**1.213e-09***	**29.715**	**5.933e-11***
		Baseline vs BMI	**19.604**	**7.927e-09***	**34.524**	**9.998e-12***	**37.511**	**3.725e-12***
		Manual vs BMI	-3.343	0.263690	**24.101**	**7.044e-10***	**12.016**	**2.145e-06***
	Gamma (35-75Hz)	Baseline vs Manual	-1.275	10.186384	**-12.249**	**1.730e-06***	-3.566	0.174739
		Baseline vs BMI	-3.511	0.193216	**-13.590**	**5.376e-07***	**-9.780**	**2.049e-05***
		Manual vs BMI	**-5.283**	**8.719e-03***	**-6.492**	**1.337e-03***	**-10.371**	**1.085e-05***
	Highgamma (75-150Hz)	Baseline vs Manual	**-19.798**	**7.067e-09***	**-9.234**	**3.785e-05***	**-19.968**	**6.396e-09***
		Baseline vs BMI	**-15.355**	**1.338e-07***	**-14.554**	**2.469e-07***	**-17.135**	**3.785e-08***
		Manual vs BMI	-0.778	20.321279	**-4.749**	**0.021287***	-1.599	6.109760
Monkey Y	Theta (4-8Hz)	Baseline vs Manual	**-46.227**	**5.844e-21***	**-57.829**	**5.493e-23***	**-8.890**	**6.564e-07***
		Baseline vs BMI	**-58.992**	**3.624e-23***	**-33.419**	**4.865e-18***	**-8.840**	**7.207e-07***
		Manual vs BMI	-1.215	10.708290	**12.666**	**1.203e-09***	**4.909**	**3.345e-03***
	Alpha (8-13Hz)	Baseline vs Manual	**-21.664**	**3.394e-14***	1.397	7.965260	**4.805**	**4.284e-03***
		Baseline vs BMI	**-8.959**	**5.759e-07***	**11.111**	**1.337e-08***	**6.093**	**2.153e-04***
		Manual vs BMI	**9.891**	**1.056e-07***	**7.638**	**7.718e-06***	0.118	40.816879
	Beta (13-35Hz)	Baseline vs Manual	**67.642**	**2.078e-24***	**31.919**	**1.254e-17***	**8.690**	**9.590e-07***
		Baseline vs BMI	**56.723**	**8.219e-23***	**33.253**	**5.389e-18***	**11.422**	**8.093e-09***
		Manual vs BMI	3.506	0.094663	1.770	4.105440	-2.659	0.660268
	Gamma (35-75Hz)	Baseline vs Manual	**33.551**	**4.485e-18***	**10.681**	**2.715e-08***	3.121	0.232506
		Baseline vs BMI	**36.025**	**1.031e-18***	0.446	29.695306	**-7.973**	**3.913e-06***
		Manual vs BMI	-3.667	0.064683	**-5.369**	**1.133e-03***	**-8.055**	**3.319e-06***
	Highgamma (75-150Hz)	Baseline vs Manual	1.343	8.718462	**5.606**	**6.544e-04***	**5.536**	**7.696e-04***
		Baseline vs BMI	-0.696	22.229325	**-5.300**	**1.334e-03***	-0.844	18.367808
		Manual vs BMI	-2.628	0.707687	**-6.402**	**1.080e-04***	**-6.064**	**2.304e-04***

Supplementary Table 1. Relevant statistics for comparing average power at the go cue between each task type for each frequency band. Paired t-test t-statistic and Bonferroni corrected p for comparison between average power normalized to total power during baseline vs manual control vs BMI control at the go cue for each monkey and frequency band. Significant differences are signified by bolded lettering and a (*).

Target: Comparing Average Power Between Task Types

Subject	Frequency Band	Comparison	M1 t-statistic	M1 Bonferroni corrected p	DLPFC t-statistic	DLPFC Bonferroni corrected p	Cd t-statistic	Cd Bonferroni corrected p
Monkey H	Theta (4-8Hz)	Baseline vs Manual	0.858	18.352398	**9.584**	**2.546e-05***	**-4.493**	**0.033084***
		Baseline vs BMI	-2.806	0.714292	3.336	0.266714	-3.038	0.464430
		Manual vs BMI	**-8.563**	**8.379e-05***	**-7.204**	**4.862e-04***	0.914	17.037005
	Alpha (8-13Hz)	Baseline vs Manual	**8.470**	**9.383e-05***	**6.627**	**1.099e-03***	**6.193**	**2.087e-03***
		Baseline vs BMI	3.812	0.111402	2.666	0.925721	**8.321**	**1.129e-04***
		Manual vs BMI	**-14.092**	**3.563e-07***	**-6.613**	**1.120e-03***	2.743	0.801803
	Beta (13-35Hz)	Baseline vs Manual	**14.504**	**2.567e-07***	**-4.276**	**0.048485***	**7.408**	**3.683e-04***
		Baseline vs BMI	**11.521**	**3.421e-06***	0.257	36.069838	**5.897**	**3.277e-03***
		Manual vs BMI	**-4.999**	**0.013939***	**5.744**	**4.162e-03***	-1.197	11.449104
	Gamma (35-75Hz)	Baseline vs Manual	**-16.978**	**4.209e-08***	**-9.743**	**2.133e-05***	**-4.371**	**0.041011***
		Baseline vs BMI	**-13.338**	**6.645e-07***	**-15.354**	**1.339e-07***	**-12.653**	**1.203e-06***
		Manual vs BMI	**11.778**	**2.679e-06***	**-7.277**	**4.403e-04***	**-11.568**	**3.271e-06***
	Highgamma (75-150Hz)	Baseline vs Manual	**-37.270**	**4.023e-12***	-3.059	0.446434	**-20.883**	**3.789e-09***
		Baseline vs BMI	**-25.543**	**3.551e-10***	**-6.223**	**1.993e-03***	**-18.878**	**1.230e-08***
		Manual vs BMI	**11.805**	**2.611e-06***	-1.006	15.038327	-2.252	1.973270
Monkey Y	Theta (4-8Hz)	Baseline vs Manual	**-12.764**	**1.040e-09***	**-4.988**	**2.772e-03***	**-5.407**	**1.038e-03***
		Baseline vs BMI	**-23.752**	**5.285e-15***	**-6.227**	**1.595e-04***	**-7.282**	**1.617e-05***
		Manual vs BMI	-3.223	0.183528	3.039	0.280638	1.291	9.481099
	Alpha (8-13Hz)	Baseline vs Manual	-2.979	0.322256	0.102	41.401543	**5.198**	**1.693e-03***
		Baseline vs BMI	**-10.931**	**1.793e-08***	-0.671	22.940993	**5.696**	**5.317e-04***
		Manual vs BMI	**-7.863**	**4.884e-06***	-0.852	18.180915	-1.953	2.890856
	Beta (13-35Hz)	Baseline vs Manual	**37.240**	**5.193e-19***	**7.426**	**1.198e-05***	**5.799**	**4.204e-04***
		Baseline vs BMI	**31.569**	**1.573e-17***	**4.780**	**4.539e-03***	**6.829**	**4.245e-05***
		Manual vs BMI	**-4.556**	**7.734e-03***	**-6.934**	**3.387e-05***	-2.866	0.416193
	Gamma (35-75Hz)	Baseline vs Manual	0.453	29.484801	2.972	0.327311	1.600	5.604214
		Baseline vs BMI	-2.029	2.489650	**-10.386**	**4.470e-08***	-3.073	0.259445
		Manual vs BMI	-1.908	3.154715	**-11.266**	**1.039e-08***	-2.883	0.400557
	Highgamma (75-150Hz)	Baseline vs Manual	**-11.982**	**3.367e-09***	**4.510**	**8.647e-03***	**5.064**	**2.321e-03***
		Baseline vs BMI	**-15.470**	**2.667e-11***	-3.389	0.124508	**6.932**	**3.404e-05***
		Manual vs BMI	**3.949**	**0.033054***	-8.501	1.380e-06	-1.918	3.095394

Supplementary Table 2. Relevant statistics for comparing average power at target acquisition between each task type for each frequency band. Paired t-test t-statistic and Bonferroni corrected p for comparison between average power normalized to total power during baseline vs manual control vs BMI control at target acquisition for each monkey and frequency band. Significant differences are signified by bolded lettering and a (*).

a

Go Cue: BMI vs Manual Target-Prediction LDA Classification Accuracy

Subject	Model Features	t-statistic	Bonferroni corrected p
Monkey H	M1	-3.114	0.106604
	DLPFC	-0.600	7.816245
	Cd	0.986	4.772351
	M1 + DLPFC	-2.555	0.320548
	**M1 + Cd**	**-3.564**	**0.043621***
	DLPFC + Cd	-0.548	8.290701
	**M1 + DLPFC + Cd**	**-3.504**	**0.049121***
Monkey Y	**M1**	**-5.744**	**3.194e-05***
	DLPFC	-1.312	2.783650
	Cd	2.604	0.194051
	**M1 + DLPFC**	**-4.775**	**5.346e-04***
	**M1 + Cd**	**-3.726**	**0.010505***
	**DLPFC + Cd**	**-0.447**	**9.214407***
	**M1 + DLPFC + Cd**	**-4.700**	**6.634e-04***

b

Target: BMI vs Manual Target-Prediction LDA Classification Accuracy

Subject	Model Features	t-statistic	Bonferroni corrected p
Monkey H	**M1**	**-9.679**	**1.955e-06***
	**DLPFC**	**-7.709**	**2.941e-05***
	Cd	-2.873	0.171915
	**M1 + DLPFC**	**-9.818**	**1.641e-06***
	**M1 + Cd**	**-8.676**	**7.362e-06***
	**DLPFC + Cd**	**-6.108**	**3.789e-04***
	**M1 + DLPFC + Cd**	**-8.616**	**8.000e-06***
Monkey Y	**M1**	**-6.609**	**2.638e-06***
	**DLPFC**	**-6.492**	**3.688e-06***
	Cd	-0.192	11.890323
	**M1 + DLPFC**	**-10.087**	**2.569e-10***
	**M1 + Cd**	**-3.962**	**5.449e-03***
	**DLPFC + Cd**	**-6.460**	**4.041e-06***
	**M1 + DLPFC + Cd**	**-7.792**	**9.661e-08***

Supplementary Table 3. Relevant statistics for comparing between *8-class* target-prediction LDA classification accuracy during BMI and manual control (a) Unpaired t-test t-statistic and Bonferroni corrected p for comparison between *8-class* target-prediction LDA classification accuracy during BMI vs manual control at go cue for each monkey and included model features. Significant differences are signified by bolded lettering and a (*). (b) Same as (a) but for target acquisition, rather than go cue.

a

Go Cue: Normalized Granger Causality vs 0

Control Type	Subject	Interaction Direction	t-statistic	Bonferroni corrected p
BMI	Monkey H	M1→DLPFC	0.183	10.294381
		**DLPFC→M1**	**6.779**	**2.353e-04***
		M1→Cd	-3.516	0.051088
		**Cd→M1**	**6.186**	**5.625e-04***
		DLPFC→Cd	-3.110	0.108243
		**Cd→DLPFC**	**6.763**	**2.409e-04***
	Monkey Y	M1→DLPFC	-0.347	8.782855
		**DLPFC→M1**	**10.463**	**1.046e-08***
		**M1→Cd**	**-9.023**	**1.362e-07***
		**Cd→M1**	**14.460**	**2.612e-11***
		**DLPFC→Cd**	**-6.417**	**2.788e-05***
		Cd→DLPFC	2.281	0.397076
Manual	Monkey H	M1→DLPFC	0.514	7.398460
		**DLPFC→M1**	**7.859**	**5.412e-05***
		M1→Cd	0.439	8.018282
		**Cd→M1**	**4.229**	**0.014034***
		**DLPFC→Cd**	**5.527**	**1.565e-03***
		**Cd→DLPFC**	**-4.282**	**0.012782***
	Monkey Y	**M1→DLPFC**	**-8.858**	**1.857e-07***
		**DLPFC→M1**	**12.398**	**4.774e-10***
		**M1→Cd**	**-7.839**	**1.367e-06***
		**Cd→M1**	**13.124**	**1.648e-10***
		**DLPFC→Cd**	**-3.873**	**0.010553***
		Cd→DLPFC	-1.447	1.951595

b

Target: Normalized Granger Causality vs 0

Control Type	Subject	Interaction Direction	t-statistic	Bonferroni corrected p
BMI	Monkey H	M1→DLPFC	0.180	10.321333
		**DLPFC→M1**	**5.573**	**1.454e-03***
		M1→Cd	-1.672	1.444127
		**Cd→M1**	**5.377**	**1.993e-03***
		DLPFC→Cd	-2.420	0.388041
		**Cd→DLPFC**	**5.418**	**1.865e-03***
	Monkey Y	M1→DLPFC	2.045	0.643538
		**DLPFC→M1**	**14.307**	**3.201e-11***
		**M1→Cd**	**-3.314**	**0.039562***
		**Cd→M1**	**11.708**	**1.374e-09***
		**DLPFC→Cd**	**-4.832**	**1.070e-03***
		**Cd→DLPFC**	**-8.625**	**2.898e-07***
Manual	Monkey H	M1→DLPFC	0.912	4.559055
		**DLPFC→M1**	**6.765**	**2.402e-04***
		M1→Cd	2.985	0.136545
		**Cd→M1**	**3.734**	**0.034255***
		**DLPFC→Cd**	**5.556**	**1.495e-03***
		**Cd→DLPFC**	**-4.828**	**4.958e-03***
	Monkey Y	M1→DLPFC	-0.514	7.349721
		**DLPFC→M1**	**9.897**	**2.785e-08***
		**M1→Cd**	**-2.701**	**0.160473***
		**Cd→M1**	**9.344**	**7.519e-08***
		**DLPFC→Cd**	**-4.613**	**1.801e-03***
		**Cd→DLPFC**	**-3.909**	**9.690e-03***

Supplementary Table 4. Relevant statistics for comparing normalized granger causality to 0 for both BMI and manual control (a) 1-sample t-test t-statistic and Bonferroni corrected p for comparison of normalized granger causality during BMI and manual control to 0 at go cue for each monkey and interaction direction. Significant differences are signified by bolded lettering and a (*). (b) Same as (a) but for target acquisition, rather than go cue.

a

Go Cue: BMI vs Manual Normalized Granger Causality

Subject	Interaction Direction	t-statistic	Bonferroni corrected p
Monkey H	M1→DLPFC	-0.235	9.818311
	DLPFC→M1	0.093	11.127620
	**M1→Cd**	**-5.681**	**1.226e-03***
	**Cd→M1**	**6.893**	**2.002e-04***
	**DLPFC→Cd**	**-9.882**	**4.882e-06***
	**Cd→DLPFC**	**10.185**	**3.525e-06***
Monkey Y	**M1→DLPFC**	**8.213**	**6.474e-07***
	DLPFC→M1	-1.463	1.898857
	M1→Cd	-0.799	5.197626
	Cd→M1	2.303	0.379286
	**DLPFC→Cd**	**-4.668**	**1.579e-03***
	Cd→DLPFC	2.813	0.125110

b

Target: BMI vs Manual Normalized Granger Causality

Subject	Interaction Direction	t-statistic	Bonferroni corrected p
Monkey H	M1→DLPFC	-1.187	3.098871
	DLPFC→M1	0.100	11.062261
	**M1→Cd**	**-6.623**	**2.947e-04***
	**Cd→M1**	**5.785**	**1.042e-03***
	**DLPFC→Cd**	**-9.106**	**1.171e-05***
	**Cd→DLPFC**	**10.515**	**2.492e-06***
Monkey Y	M1→DLPFC	2.743	0.146179
	**DLPFC→M1**	**5.388**	**2.896e-04***
	M1→Cd	-1.366	2.234999
	Cd→M1	1.531	1.689782
	DLPFC→Cd	-1.275	2.596613
	**Cd→DLPFC**	**-3.646**	**0.018125***

Supplementary Table 5. Relevant statistics for comparing between normalized granger causality during BMI and manual control (a) Paired t-test t-statistic and Bonferroni corrected p for comparison between normalized granger causality during BMI vs manual control at go cue for each monkey and interaction direction. Significant differences are signified by bolded lettering and a (*). (b) Same as (a) but for target acquisition, rather than go cue.

a

Go Cue: Net Normalized Granger Causality vs 0

Control Type	Subject	Interaction Direction	Net Normalized GC	SEM Across Days	t-statistic	Bonferroni corrected p
BMI	Monkey H	**DLPFC→M1**	**0.737**	**0.137**	**5.392**	**9.734e-04***
		**Cd→M1**	**1.294**	**0.226**	**5.715**	**5.810e-04***
		**Cd→DLPFC**	**0.383**	**0.082**	**4.672**	**3.236e-03***
	Monkey Y	**DLPFC→M1**	**0.088**	**0.017**	**5.304**	**1.762e-04***
		**Cd→M1**	**0.232**	**0.019**	**12.167**	**3.386e-10***
		**Cd→DLPFC**	**0.157**	**0.019**	**8.456**	**2.008e-07***
Manual	Monkey H	**DLPFC→M1**	**0.728**	**0.111**	**6.556**	**1.624e-04***
		Cd→M1	0.535	0.192	2.791	0.097935
		**Cd→DLPFC**	**-0.605**	**0.113**	**-5.366**	**1.015e-03***
	Monkey Y	**DLPFC→M1**	**0.163**	**0.013**	**12.205**	**3.196e-10***
		**Cd→M1**	**0.210**	**0.019**	**11.314**	**1.283e-09***
		Cd→DLPFC	0.033	0.015	2.228	0.221623

b

Target: Net Normalized Granger Causality vs 0

Control Type	Subject	Interaction Direction	Net Normalized GC	SEM Across Days	t-statistic	Bonferroni corrected p
BMI	Monkey H	**DLPFC→M1**	**0.562**	**0.126**	**4.457**	**4.701e-03***
		**Cd→M1**	**0.987**	**0.221**	**4.473**	**4.573e-03***
		**Cd→DLPFC**	**0.297**	**0.087**	**3.402**	**0.031479***
	Monkey Y	**DLPFC→M1**	**0.094**	**0.015**	**6.177**	**2.379e-05***
		**Cd→M1**	**0.147**	**0.022**	**6.841**	**5.521e-06***
		Cd→DLPFC	**-0.032**	**0.016**	-2.044	0.322138
Manual	Monkey H	**DLPFC→M1**	**0.539**	**0.104**	**5.179**	**1.379e-03***
		Cd→M1	0.233	0.194	1.200	1.520397
		**Cd→DLPFC**	**-0.633**	**0.115**	**-5.496**	**8.231e-04***
	Monkey Y	**DLPFC→M1**	**0.083**	**0.015**	**5.465**	**1.210e-04***
		**Cd→M1**	**0.121**	**0.021**	**5.739**	**6.424e-05***
		Cd→DLPFC	0.006	0.014	0.435	4.008821

Supplementary Table 6. Relevant statistics for comparing net normalized granger causality to 0 for both BMI and manual control (a) Net normalized Granger causality values and standard error mean (SEM) across days at go cue as well as t-statistic and Bonferroni corrected p from 1-sample t-tests comparing net normalized granger causality

during BMI and manual control to 0 at go cue for each monkey and interaction direction. Significant differences are signified by bolded lettering and a (*). (b) Same as (a) but for target acquisition, rather than go cue.
